# Simultaneous extraction and preliminary purification of polyphenols from grape pomace using an aqueous two-phase system exposed to ultrasound irradiation: Process characterization and simulation

**DOI:** 10.3389/fnut.2022.993475

**Published:** 2022-11-14

**Authors:** Guangjie Xie, Juan Shen, Ji Luo, Dandan Li, Yang Tao, Changnian Song, Yongbin Han

**Affiliations:** ^1^Whole Grain Food Engineering Research Center, College of Food Science and Technology, Nanjing Agricultural University, Nanjing, China; ^2^Institute of Agricultural Products Processing, Jiangsu Academy of Agricultural Sciences, Nanjing, China; ^3^College of Horticulture of Nanjing agricultural University, Nanjing, China

**Keywords:** ultrasound, aqueous two-phase, grape pomace, phenolic compounds, artificial neural network, diffusion model, purification

## Abstract

In this study, an ultrasound-assisted aqueous two-phase (ATP) extraction method was used for the extraction and purification of phenolic compounds from grape pomace. The effect of acoustic energy densities (AED, 41.1, 63.5, 96.1, 111.2 W/L) and temperatures (20, 30, 40°C) on the yield of phenolics was investigated. An artificial neural network (ANN) was successfully used to correlate the extraction parameters with phenolic yield. Then, a diffusion model based on Fick's second law was used to model the mass transfer process during ultrasound-assisted ATP extraction and evaluate the effective diffusion coefficient of phenolics. The results revealed the increase in AED, and the temperature increased the effective diffusivity of phenolics. The HPLC analysis of anthocyanins and flavonols showed that ultrasound significantly increased the extraction yield of anthocyanins compared with the traditional method. High amounts of rutin and myricetin were recovered using the ATPS systems. Sugars were mainly distributed in the bottom phase, whereas phenolics were located in the top phase. Conclusively, ultrasound-assisted aqueous two-phase (ATP) extraction can be used as an effective method to achieve the simultaneous separation and preliminary purification of phenolics from grape pomace.

## Introduction

Grape pomace is a major byproduct of the grape processing industry. It mainly comprises peel, stem, seed, and part of pulp (1). The amounts of waste and byproducts of grapes make up ~20% of the total processed grapes (2). Currently, the utilization of grape pomace is low, and more studies are needed to explore the strategies for the utilization of grape pomace. Grape pomace is rich in bioactive phenolic compounds, which exert antiproliferative properties against colon cancer cells (3). Moreover, phenolics have antioxidant, antibacterial, cardioprotective, and skin anti-aging activities (4–8). Therefore, grape pomace can serve as raw materials for the recovery of phenolics for effective resource utilization and increment of economic value.

Several methods for the extraction of phenolic compounds have been explored, such as alkaline hydrolysis treatment (9), solvent extraction and pressurized liquid extraction (10), enzyme-assisted extraction (11), and microwave-assisted extraction (12). The methods mentioned earlier have certain problems, such as low extraction rate, environmentally unfriendly, and high energy consumption. However, ultrasound-assisted extraction (UAE) is a novel method for the extraction of phenolics. UAE improves the extraction rate and extraction yield of active ingredients (13). Meanwhile, aqueous two-phase extraction (ATPE) is widely applied in the separation of biomolecules (14). For example, ethanol coupled with ammonium sulfate is a common and economic aqueous two-phase system (ATPS) used for the extraction of anthocyanins from mulberry (15). ATPE method is used for the extraction and purification of active compounds, thus improving the purity of the extract. The combination of the two strategies results in efficient separation of bioactive substances from the material. Therefore, the combined ultrasound and ATPE approach can ensure simultaneous efficient extraction and purification (16). In the literature, ATPE was combined with the ultrasonic approach as an ultrasound-assisted aqueous two-phase method to extract polyphenols from the chaff (17). However, the research on the purification of bioactive compounds using this method is still limited. Thus, grape pomace can be used with high value, and it provides a new method for preliminary separation and purification for industrial production to study the recovery of polyphenols from grape pomace using the method of ultrasound-assisted aqueous two-phase extraction.

An artificial neural network (ANN) is a method of data analysis designed to simulate the function of the human brain. This method has been widely applied in the food-science discipline (18–20). ANN has been utilized to simulate and optimize complex food processes (21). For example, Tao et al. studied the influences of various operating parameters on the extraction yield of phenolics from wine lees (22). Tao et al. also studied the optimization of encapsulation of blueberry anthocyanin extracts by an artificial neural network and genetic algorithm (23). Furthermore, the construction of mathematical models with physical significance can be used to study mass transfer mechanisms during extraction. Such a numerical simulation method based on a diffusional model was employed to clarify the effect of ultrasound on the mass transfer mechanism during yeast biosorption (20). Besides, the physical simulation can be helpful to visualize and explore the mass transfer mechanism of extraction (24). However, few studies have used physical models to explore the effect of ultrasound-assisted ATP extraction of phenolics.

In the present study, ultrasound was combined with aqueous two-phase (ATP) for the simultaneous extraction and purification of phenolic substances from grape pomace. The aqueous two-phase system (ATPS) was established, and the effects of acoustic energy density, temperature, and ultrasound duration on the yield of total phenolics were explored. An artificial neural network model was used to explore the relationship between extraction parameters and the yield of phenolics. Moreover, the extraction mechanism was established using the diffusion model. The profiles of individual phenolics in the top and bottom phases were analyzed by HPLC. In addition, the change in sugar content in the system was evaluated, and the purity of phenolic extract was determined. This study aimed to establish an appropriate and easy extraction and purification strategy for phenolic substances from grape pomace, as well as to provide a theoretical basis and technological guidance for the extraction process.

## Materials and methods

### Materials and chemicals

Seedless grapes with similar maturity (Cultivar: Xiahei) were purchased from Nanjing Zhongcai Agricultural Product Market (Jiangning, Nanjing). After reaching the laboratory, grape samples were immediately stored at −20°C in darkness before use. Ammonium sulfate and anhydrous ethanol were purchased from Sinopharm Chemical Reagents Co., Ltd. (Shanghai, China). All other chemicals used were of analytical or chromatographic grades.

### Preparation of grape pomace

Frozen grapes were thawed at room temperature, washed, and pressed using a domestic juice extractor purchased from Midea Household Appliance Manufacturing Co., Ltd (MJ-WBL2501A, China). Grape pomace was collected, dried at 50°C for 12 h, grounded, and sieved to obtain particles with the size between 425 and 125 μm. The grape pomace samples were stored at −20°C in darkness.

### Establishment of an aqueous two-phase system

ATPS comprising ethanol and ammonium sulfate was selected as the solvent for the extraction of phenolics from grape pomace, according to previous studies (25, 26).

**Figure 1:**
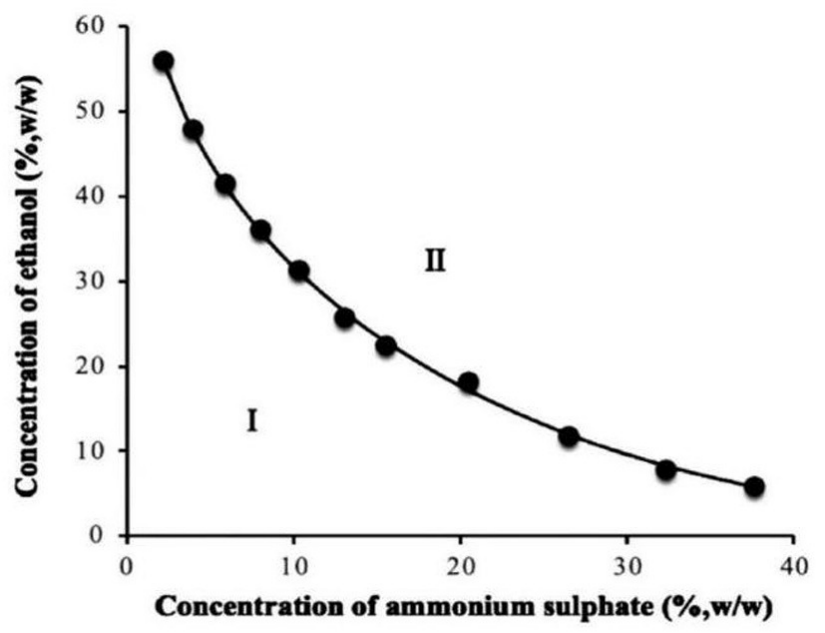
Phase diagram for ethanol-ammonium sulfate aqueous two-phase system.

The formation of ethanol-ammonium sulfate ATP mainly involves the competition between alcohol and salt for water molecules. The system has divided the suspensions into two phases when the two sides compete for water to a certain extent due to the repulsion, namely the ethanol-rich phase and ammonium sulfate-rich phase (27). The phase diagram of ethanol-ammonium sulfate measured in this study is presented in Figure 1. The region above the curve is the two-phase region (II). The two phases are formed when alcohol and salt reach a certain proportion. In this region, the top phase is rich in alcohol and the bottom phase is rich in salt.

Two aqueous phase system was established based on the phase diagram. The operation steps of the two-phase system construction: dissolve a certain mass of ammonium sulfate in a certain volume of deionized water, and then add a certain volume of ethanol to make the total mass of the two-phase system (100 g). Stir until the solution is clarified. After standing for 1 min, the system is divided into two phases: the upper phase is the ethanol-rich phase and the lower phase is the salt-rich phase.

The two-node data were fitted using the empirical equation of binodal curves suitable for small molecular alcohol-salt ATPS (28). The equation for the binodal curve is expressed as follows:


(1) $$\begin{array}{l} w_{1}=\exp\left(a+bw_{2}^{0.5}+cw_{2}+dw_{2}^{2}\right) \end{array}$$

where *w*_1_ represents the mass fraction of ethanol, *w*_2_ represents the mass fraction of ammonium sulfate; and *a, b, c*, and *d* represent the fitting parameters. The data were calculated and fitted using MATLAB R2016a. The values of the relevant parameters were then obtained. The following relationship was obtained after substituting the parameters:


(2) $$\begin{array}{l} w_{1}=exp\left(-0.1408-2.882w_{2}^{0.5}-0.3998w_{2}-5.608w_{2}^{2}\right) \end{array}$$

The correlation coefficient *R*^2^ obtained by fitting is 0.9992 and the absolute error *AAD* value is 2.02%. Notably, *R*^2^ is close to 1 and the *AAD* value is <5% indicating a good fitting degree. The concentrations of ethanol and ammonium sulfate required for phase equilibria can be effectively obtained using this equation, thus facilitating the construction of ATPS. In addition, the phase diagram of ATPS provides a theoretical basis for the design of subsequent extraction experiments.

Xavier et al. (26) reported that phenolics were mainly distributed in the top phase and recovered in the top phase in the ATPS. Using the recovery and yield of phenolics as the index, the concentration of ethanol and ammonium sulfate was screened at 30°C and 96.1 W/L to determine the appropriate concentration of phase for phenolics extraction. According to the phase diagram, the ATP solvent consists of ethanol and ammonium sulfate.

### ATP-assisted simultaneous extraction and preliminary purification under ultrasonic irradiation

First, 2.5 g of grape pomace particles were added into a 100 mL conical flask containing 50 mL aforementioned ATP solution. Then, this flask was fixed well in a thermostatic water bath system. The 20-kHz ultrasound probe was inserted into the solution at the same depth of 2 cm. The ultrasound probe was connected with an ultrasound generator (VCX130, Sonics and Materials Inc., Newtown, USA). The actual acoustic energy density (AED) distributed in the ATP solution corresponding to a certain ultrasonic power was measured by the calorimetry method (29). The detailed experimental setup is illustrated in Supplementary Figure 1. Ultrasonic treatment was performed in an intermittent mode of 5 s on and 5 s off. During sonication, the samples in both upper and lower phases were taken periodically.

### Experimental design

A full-factorial design was employed to generate the experimental runs, to explore the effects of AED levels (41.1, 63.5, 96.1, 111.2 W/L), temperature (20, 30, 40°C), and time on the yield of phenolics. For comparison purpose, the ATP extraction test under reciprocating shaking at 30°C and 100 rpm was taken as a control. All the processes were performed in triplicates.

### Chemical analysis

#### Determination of total phenolic content

Total phenolic content was determined by the well-known Folin-Ciocatteu method (26), which was expressed as gallic acid equivalent in mL. The detailed procedure was described in the study of Singleton et al. (25). The distribution coefficient and recovery of polyphenols are calculated using the following equations:


(3) $$\begin{array}{l} K=\frac{C_{T}}{C_{B}} \end{array}$$

(4) $$\begin{array}{l} Y_{T}\left(\%\right)=\frac{C_{T}V_{T}}{\left(C_{T}V_{T}+C_{B}V_{B}\right)}\times 100\% \end{array}$$

(5) $$\begin{array}{l} Y_{B}\left(\%\right)=\frac{C_{B}V_{B}}{\left(C_{T}V_{T}+C_{B}V_{B}\right)}\times 100\% \end{array}$$

where *Y*_*T*_ represents the recovery of polyphenols in the top phase (%), *Y*_*B*_ indicates the recovery of polyphenols in the bottom phase (%), *C*_*T*_ represents the total phenolic concentration in the top phase (mg/L), and *C*_*B*_ indicates the total phenolic concentration in the bottom phase (mg /L). *V*_*T*_ and *V*_*B*_ indicate the volumes of the top phase solution and bottom phase solution, respectively.

#### Determination of total sugar content

The concentration of total sugar was determined by Anthrone sulphuric acid colorimetry (30). Specifically, 1 mL of the sample was mixed with 4 mL of sulfuric acid anthrone reagent (2 mg/mL). After shaking, the mixture was quickly placed in a boiling water bath for 10 min and then cooled in an ice water bath for 10 min. The absorbance at 620 nm was measured. Glucose was used as the standard to make the calibration curve and the content was expressed as mg glucose/mL.

#### Determination of purity of phenolic samples

The top phase solution (containing phenolics) after ATP extraction was collected and the involved phenolic amount was determined. Then, the samples were desalted by run water dialysis (15). The extract was concentrated by rotational evaporation. The samples were freeze-dried for 72 h and weighed. The purity of the samples was determined as the ratio of the total phenolic mass to the extracted mass.

#### Analysis of phenolic profile

HPLC (LC-2010A, Shimadzu, Japan) was used to explore the profile of individual phenolics obtained from ultrasonic-assisted ATP extraction, water bath oscillation ATP extraction, and crude extraction using 50% ethanol solution, as well as individual phenolics in grape pomace. Contents of individual anthocyanins were determined using HPLC as described previously (31). Chromatographic conditions and methods were slightly modified. The sample was analyzed by HPLC after passing through a 0.45 μm organic filtration membrane. An Agilent TC-C18 column (250 × 4.6 mm, 5 μm) was used for the separation of anthocyanins. The mobile phase comprised (A) trifluoroacetic acid (0.5%) and (B) pure acetonitrile solution. A gradient elution program was used as follows: 0 ~ 5 min, 15 ~ 18% B; 5 ~ 11 min, 18 ~ 21% B; 11 ~ 13 min, 21 ~ 22% B; 13 ~ 15 min, 22 ~ 23% B; 15 ~ 19 min, 23 ~ 24% B; 19 ~ 22 min, 24 ~ 25% B; 22 ~ 35 min, 25 ~ 30% B; 35 ~ 45 min, 15% B. The column temperature and detection wavelength were 30°C and 520 nm, respectively. Injection volume and the flow rate were set at 10 μL and 0.6 mL/min, respectively.

Contents of flavonols were determined by HPLC following a method described previously (32). The chromatographic conditions and methods were slightly modified. Pretreatment was conducted before analysis (33). The pH value of the polyphenol extract was first adjusted to 7.0. Extraction was then performed with ethyl acetate at a volume ratio of 1:1. The extraction time was 20 min, and the extraction was conducted in triplicates. After each extraction, the organic phase was collected, and the extract was concentrated by rotational evaporation at 40°C. The extract was then dissolved in methanol. The sample was analyzed by HPLC after passing through a 0.45 μm organic filtration membrane. The chromatographic conditions included a mobile phase comprising (A) acetic acid aqueous solution (1%) and (B) methanol acetate solution (1%). A gradient elution program was used as follows: 0–10 min, 10–26% B; 10–25 min, 26–40% B; 25–45 min, 40–65% B; 45–55 min, 65–95% B; 55–58 min, 95–10% B; 58–65 min, 10% B. Column temperature and detection wavelength were set at 25°C and 350 nm, respectively. Injection volume and the flow rate were set at 20 μL and 0.6 mL/min, respectively. The content was expressed as mg/g.

#### Determination of particle size

The particle size of grape pomace before and after extraction was determined by a laser particle size analyzer [LS-C(III), OMEC, Zhuhai, China]. The obscuration rate, particle refraction index, and particle absorption rate were set as 5–10%, 1.57, and 0.001, respectively.

### Mathematical modeling

#### Artificial neural network model of extraction parameters

The correlation between ATPE parameters and the phenolic extraction yield was explored using a statistical model. For this purpose, a three-layer feedforward artificial neural network model was established in the present study. The model mainly comprised an input layer, hidden layer, and output layer, with several neurons. AED, temperature, and time represented three nodes of the input layer, and the total phenol yield was included in a neuron of the output layer. The number of neurons in the hidden layer was adjusted from 5 to 20, and the transfer function type was also tested (34).

Transfer functions used in neural network model construction mainly included logsig function, tansig function, and purelin function. The coefficient of determination *R*^2^, root mean square error *RMSE*, and absolute error *AAD* were determined to evaluate the constructed model. These parameters were determined as follows (35):


(6) $$\begin{array}{l} R^{2}=1-\frac{\sum_{i=1}^{n}{\left(Y_{i,p}-Y_{i,e}\right)}^{2}}{\sum_{i=1}^{n}{\left(Y_{i,e}-Y_{m}\right)}^{2}} \end{array}$$

(7) $$\begin{array}{l} RMSE=\sqrt{\frac{\sum_{i=1}^{n}{\left(Y_{i,e}-Y_{i,p}\right)}^{2}}{n}} \end{array}$$

(8) $$\begin{array}{l} AAD=\left[\frac{\sum_{i=1}^{n}\left(|Y_{i,p}-Y_{i,e}|/Y_{i,e}\right)}{n}\right]\times 100 \end{array}$$

where *Y*_*i,p*_ indicates the predicted extraction yield of the test model (mg/g), *Y*_*i,e*_ represents the experimental value (mg/g), *Ym* indicates the average experimental value (mg/g), and n represents the number of test groups.

The neural network toolbox in MATLAB r2016a was used for the construction of the artificial neural network model. The model can be expressed as follows:


(9) $$\begin{array}{l} Y_{ANN}=purelin\left(LW^{\left(2,1\right)}tansig\left(IW^{\left(1,1\right)}xn+b^{\left(1\right)}\right)+b^{\left(2\right)}\right) \end{array}$$

where *Y*_*ANN*_ represents the extraction yield of phenolics under a certain condition predicted by the model, *LW*^(2,1)^ indicates the layer weight matrix, *IW*^(1,1)^ represents the input weight matrix, *x*_*n*_ represents the input test condition, *b*^(1)^ indicates the target deviation of the hidden layer, and *b*^(2)^ represents the target deviation of the output layer.

#### Mass transfer diffusion model

A diffusion model based on Fick's second law was used to simulate the intraparticle diffusion process of phenolic during the extraction process. This simulation was then used to explore the mechanism of ultrasonic-enhanced phenolics extraction in the entire aqueous two-phase system, to determine the effective diffusion coefficient, as well as to study the promotion effect of ultrasound. The following assumptions were considered:

(i) Grape pomace particles are spherical, and phenolic compounds were uniformly distributed in the particles before extraction, and the content only varied with time and space.
(ii) Chemical reactions and degradation of phenolic compounds did not occur during the entire extraction process.
(iii) Distribution of polyphenols in ATP solution during sonication was uniform.

The equation of the spherical diffusion model of phenolic compounds is shown below (36):


(10) $$\begin{array}{l} \frac{\partial C_{s}}{\partial t}=D_{e}\left(\frac{1}{x^{2}}\frac{\partial }{\partial x}\left(X^{2}\frac{\partial C_{s}}{\partial x}\right)\right) \end{array}$$

where Cs represents the distribution content of phenolics; *De* indicates the effective diffusion coefficient of phenolics; *x* represents the radial distance of phenolics diffusion direction; t indicates the diffusion time.

The initial and boundary conditions of the diffusion model were as follows:


(11) $$\begin{array}{l} C_{s}\left(x,0\right)=C_{S}^{0}\;0\leq x\leq r \end{array}$$

(12) $$\begin{array}{l} C_{L}\left(0\right)=0 \end{array}$$

The following equation can be obtained from Equations (10, 11):


(13) $$\begin{array}{l} -D_{e}A\left[\frac{\partial C_{s}\left(x,t\right)}{\partial x}\right]=V\frac{dC_{L}}{dt}\;x=r \end{array}$$

where *r* represents the radius of the particle (m), *C*_*L*_ indicates the average concentration of phenolics in the ATP solution (g/m^3^), *A* represents the surface area of the grape pomace particle (m^2^), and *V* indicates the volume of the extraction solution (m^3^).

Equation (10) was solved using the *pdepe* function in Matlab2016a, and the initial and boundary conditions were determined. The diffusion coefficient *De* was adjusted until the *RMSE* value between the predicted value and the experimental value of extraction yield was minimized. Then, *R*^2^ and *AAD* values were used as model evaluation indexes.

### Statistical analyses

All treatments and analyses were conducted in triplicate. Data were expressed as mean ± SD values. Statistical analysis was performed using Microsoft Office 2013 and SAS version 9.2 (SAS Institute, Cary, NC, USA). Duncan's multiple comparison method was used to compare mean values among groups. *P* < 0.05 represented statistical significance. MATLAB2016a was used to analyze and determined the correlation between the artificial neural network model and the mass transfer and diffusion model.

## Results and discussion

### Determination of the phase composition and concentration

**Figure 2:**
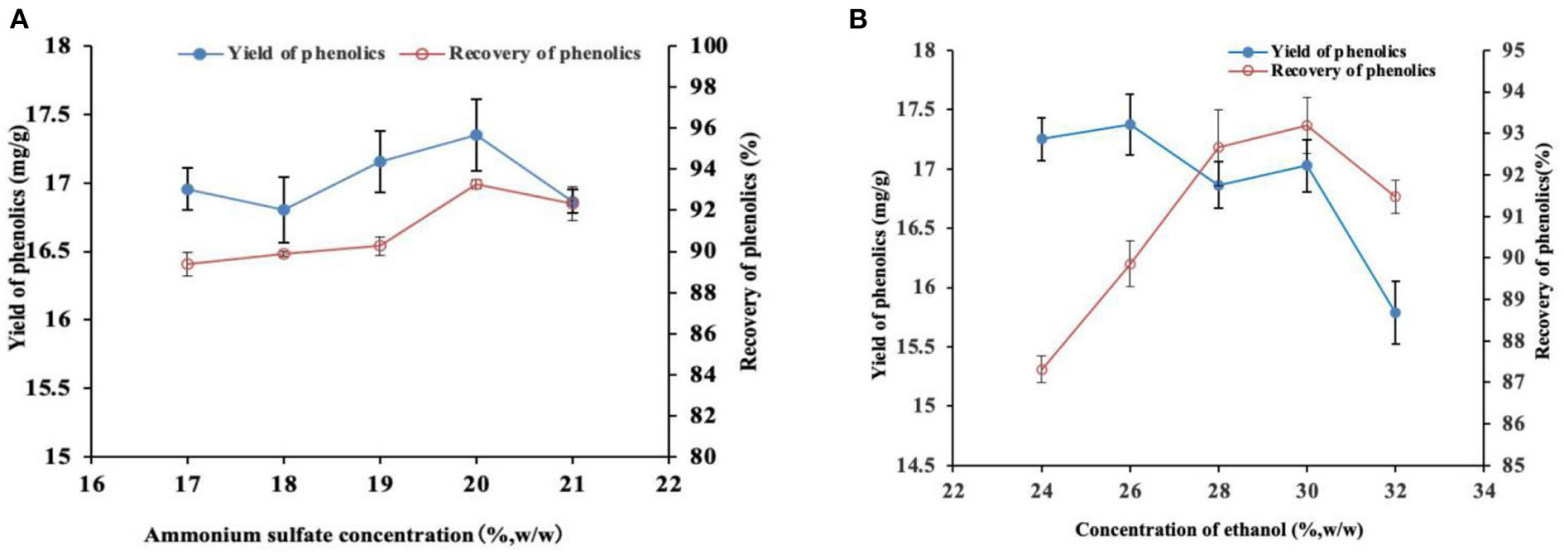
Effect of different ammonium sulfate **(A)** and ethanol **(B)** concentrations on the yield and recovery of phenolics.

Ultrasound-assisted ATPE was then performed at 40°C and 96.1 W/L for 60 min to determine the concentrations of ammonium sulfate and ethanol. The phase diagram and relevant literature (15) indicated that when the concentration of ethanol was 30% (w/w) and the concentration of ammonium sulfate was in a certain range, the ATPS was separated stably. Therefore, the concentration of ethanol was fixed at 30%(w/w) and the concentration of ammonium sulfate was then determined. The results for the determination of ammonium sulfate concentration are presented in Figure 2A. Recovery of phenolics was highest at 93.27% when the concentration of ammonium sulfate was 20% (Figure 2A). This yield was significantly higher relative to that under other concentrations (*P* < 0.05). Notably, the highest yield achieved at a 20% concentration was not significantly different compared with the extraction when the concentration of ammonium sulfate was 21% (*P* > 0.05). The extraction yield of the target product is another basic index to evaluate the performance of ATPS. The phenolic extraction yield at 20% ammonium sulfate was 17.35 mg/g, which was higher compared with that under other ammonium sulfate concentrations. However, the difference was not statistically significant (*P* > 0.05). Therefore, the 20% ammonium sulfate concentration was selected in the subsequent tests based on the results of the two evaluation indexes.

The concentration of ammonium sulfate was fixed at 20%, and the optimum concentration of ethanol was determined based on the recovery and yield of phenolics. The phenolic recovery was the highest (93.19%) at 30% ethanol concentration, which was significantly higher compared with other concentrations (Figure 2B). However, the optimum recovery was not significantly different compared with the recovery at the ethanol concentration of 28% (*P* < 0.05). The phenolic yield was highest (17.37 mg/g) at 26% ethanol concentration, which was significantly higher relative to that achieved at 32% ethanol concentration. However, the optimum yield was not significantly different relative to that achieved at other ethanol concentrations (*P* > 0.05). Therefore, the 30% ethanol concentration was selected in the subsequent experiments. In summary, the ethanol-ammonium sulfate ATPS has good distribution and extraction performance at 30% ethanol concentration and 20% ammonium sulfate concentration. Accordingly, ATPS was established based on these concentrations of the phase components for the following research.

### Comparison of ultrasound-assisted and reciprocating shaking extraction

**Figure 3:**
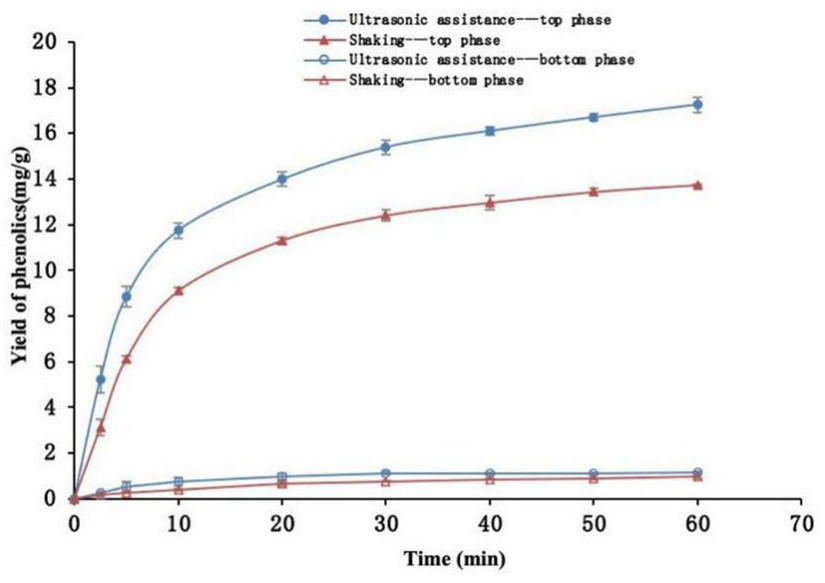
Phenolics yield after extraction with and without ultrasonic assistance.

Variation of phenolic yield with extraction time under ultrasonic-assisted (30°C, 96.1 W/L) and reciprocating shaking (30°C, 100 rpm) conditions was compared as shown in Figure 3. The findings indicated that the yields of phenolics in the top and bottom phases rapidly increased at the first 10 min and then slowly increased after this point. The yield of phenolics in the top phase was higher compared with that in the bottom phase throughout the extraction period. The distribution coefficient of phenolics in the top phase at 60 min an extraction time was 12.23 and the recovery was 93.70% under ultrasonic conditions. The distribution coefficient at this extraction period was 12.12 and the recovery was 93.40% under the reciprocating shaker conditions. The results showed no significant difference in distribution coefficient and recovery between the two extraction methods (*P* > 0.05). The distribution coefficient of phenolics in ethanol solution was significantly higher relative to that in the salt solution, implying that they were mainly distributed in the top phase solution (37).

The results showed that the phenolic yield with ultrasound-assisted was significantly higher compared with that with reciprocating shaker (*P* < 0.05) throughout the extraction process. The phenolic yield with ultrasound-assisted increased by 25.12% when the extraction time was 60 min compared with the yield with reciprocating shaking. Notably, the ultrasonic wave had a significant effect on improving the extraction efficiency of phenolics. Ultrasonic wave significantly increases the extraction yield of phenolics compared with reciprocating shaking mainly by improving the diffusion of phenolics from grape pomace particles to the solvent (38).

### Effects of AED and temperature on yield of phenolics during extraction

**Figure 4:**
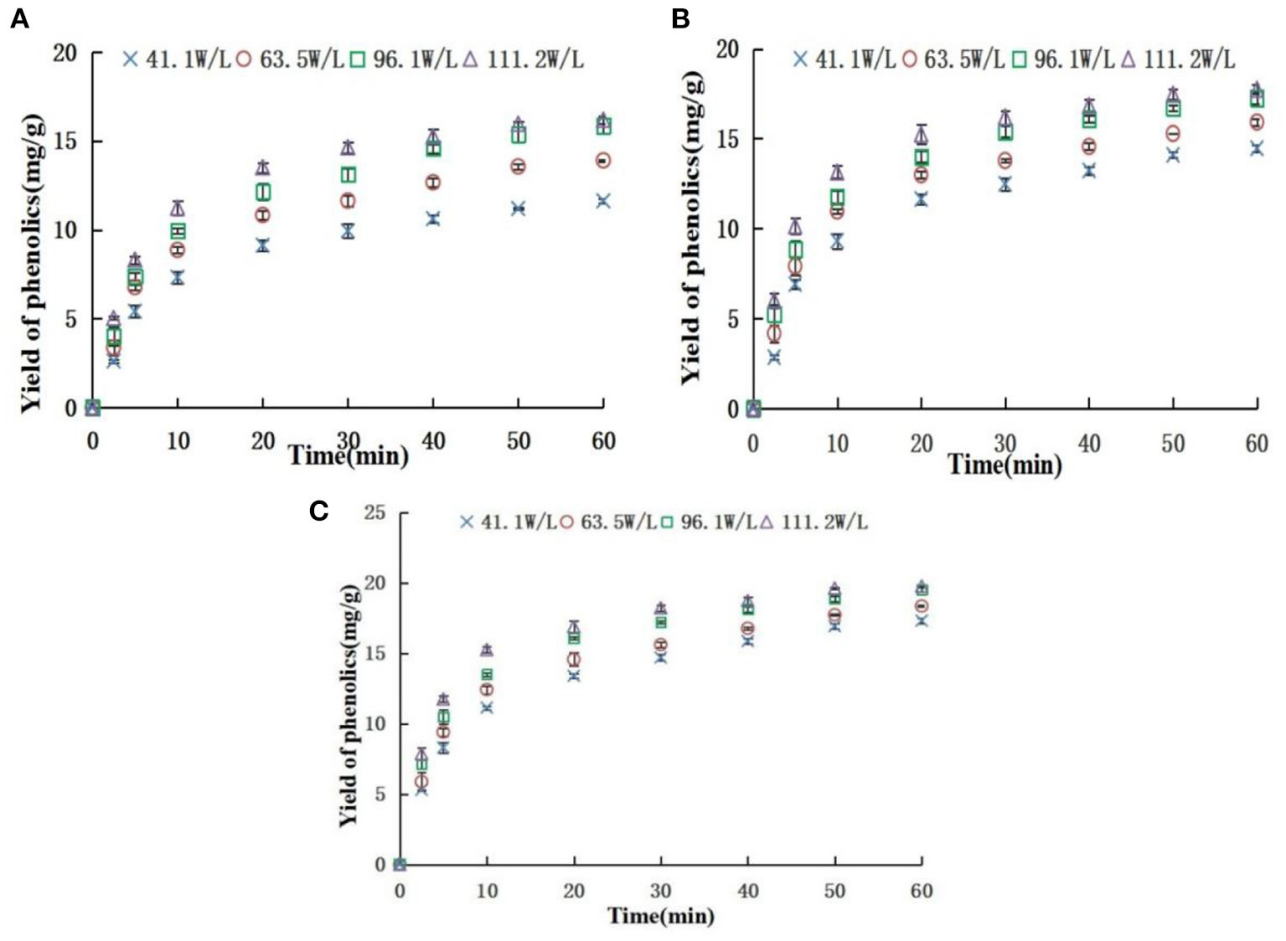
Effect of acoustic energy density on yield of phenolics [**(A)** 20°C; **(B)** 30°C; **(C)** 40°C].

The yield of the phenolics in the top phase was used as an index to explore the effect of ATP extraction parameters. Effects of different AED on the yield of phenolics at 20, 30, and 40°C are shown in Figure 4. The findings showed that the yields of phenolics rapidly increased at the first 20 min and then slowly increased under the same temperature and different AEDs, and approached equilibrium at 60 min. The yield of phenolics increased with an increase in AED. AED had a significant effect on phenolic yield (*P* < 0.05). This implies that an increase in AED promotes surface washing of phenolics and unimpeded diffusion of the broken particle solute in the early stage, thus increasing extraction yield. Moreover, an increase in AED in the internal diffusion stage where the phenolics were impeded may promote breakage of the plant cell wall and accelerate the release of phenolics, thus increasing the extraction yield of phenolics. The findings from the preliminary experiment showed that the acoustic energy density within the scope of this study did not cause significant degradation of phenolics. However, previous studies report that a significantly high AED can promote the degradation of extracted components (39). In addition, the energy resulting from the bursting of cavitation bubbles is reduced when the AED reaches a certain level, ultimately reducing the promotion effect on extraction (40). Therefore, a reasonable ultrasonic intensity should be selected for practical application.

**Figure 5:**
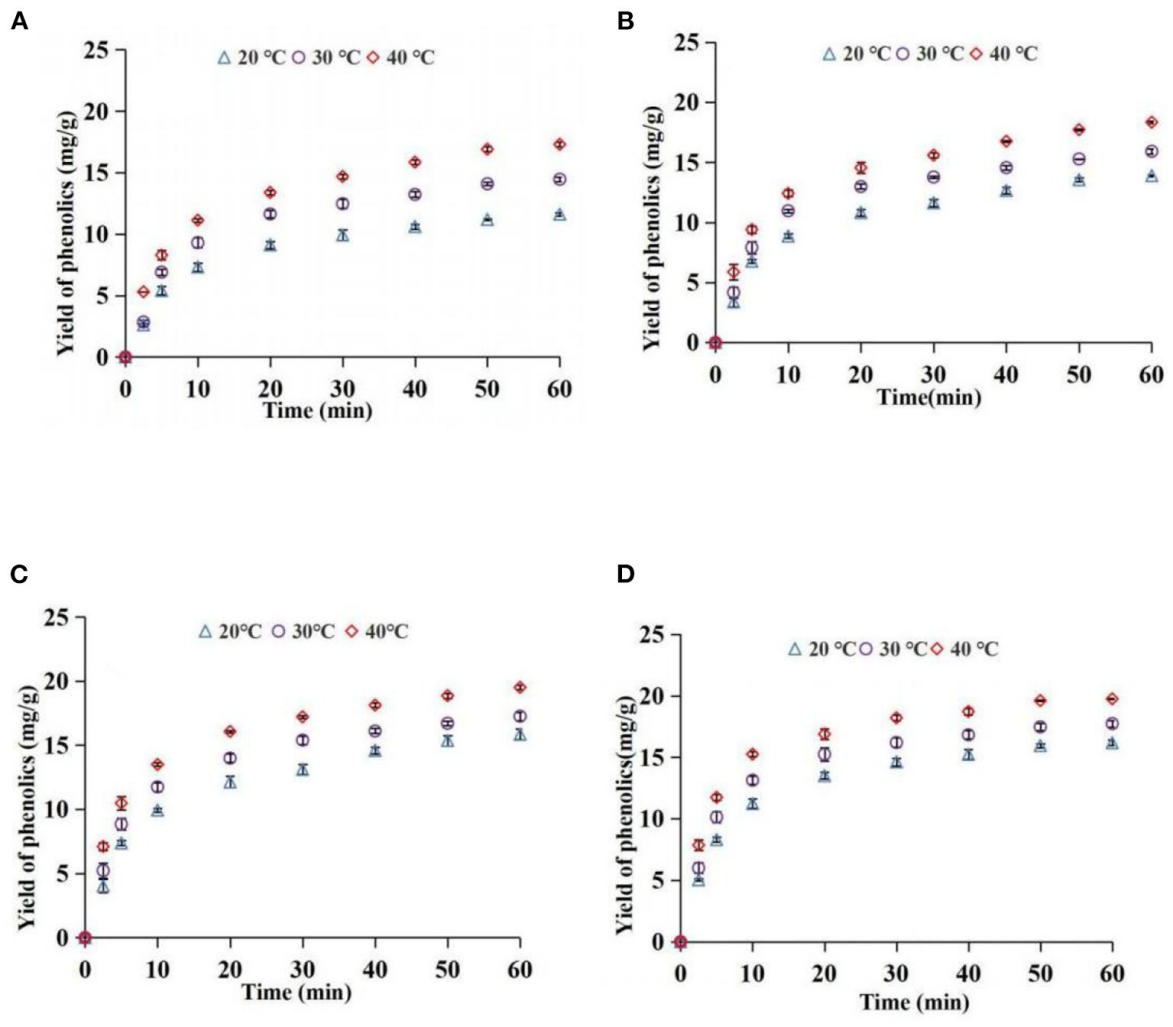
Effect of temperature on yield of phenolics [**(A)** 41.1 W/L; **(B)** 63.5 W/L; **(C)** 96.1 W/L; **(D)** 111.2 W/L].

Effects of different temperatures on the yield of phenolics at 41.1, 63.5, 96.1, and 111.2 W/L are presented in Figure 5. The yield of phenolics increased with an increase in extraction time at different temperatures. A higher temperature was associated with a higher yield of phenolics compared with lower temperatures (*P* < 0.05) at the same extraction time. An increase in temperature increased solubility and diffusion coefficient of phenolics and promoted softening and swelling of particles, as well as reduced the viscosity of the solvent. These factors promote the mass transfer of phenolic substances in the system (41). Notably, the excessive temperature had a negative effect on extraction yield. Phenolics are heat-sensitive compounds, which are easily degraded at extremely high temperatures. In addition, extremely high temperatures reduce the energy generated when ultrasonic cavitation bubbles burst (24). Therefore, a suitable temperature should be selected for the extraction of phenolics.

### Establishment of artificial neural network model

The topology of the model was optimized by adjusting the number of neurons in the hidden layer. The number of neurons in the hidden layer in the present study was eight. Components of the neural network used in this study are presented in Supplementary Figure 2. Regression of predicted values obtained after data training, verification, and testing is presented in Supplementary Figure 3. The correlation coefficient *R*-value was above 0.997. These findings and the dispersion degree of data distribution showed that the regression of the model obtained after training was satisfactory (22).

Optimal parameters and fitting performance evaluation results of the artificial neural network model are presented in Table 1. The findings showed that the correlation coefficient *R*^2^ of this model was high (0.998), whereas the *RMSE* and *AAD* values were low (0.211 mg/g and 1.846%, respectively). This implies that the model fitting and prediction values were satisfactory. Theoretically, this algorithm can predict the yield of phenolics under any conditions within the range of the experimental conditions. Therefore, it can be used for effective visualization and intelligent extraction of phenolics.

**Table 1:** Model-related parameters and fitting performance evaluation results.

**Input weight matrix** **(destination: hidden layer;** **source: inputs)**			**Bias vector** **(destination: hidden layer)**	**Layer weight matrix** **(destination: output layer;** **source: hidden layer)**		**Bias** **(destination: output layer)**	***R^2^***	**RMSE**	**ADD**
−0.0208	−1.8692	0.4737	3.657	−0.8835	T				
−0.5234	−0.232	−0.3317	1.9563	−1.2199					
−0.0265	−0.0456	4.5969	5.4388	2.3043					
0.1863	0.2145	0.7919	0.4626	0.6161		−1.0773	0.998	0.211	1.846
0.3811	0.4395	−0.2017	1.4884	1.1					
−0.9496	−4.0299	−7.4196	4.7981	0.0043					
1.0147	−1.9058	−1.1701	−0.5057	0.0354					
4.7435	2.6728	−0.2655	4.7459	−0.0408					

### An extraction diffusion model was successfully established

**Figure 6:**
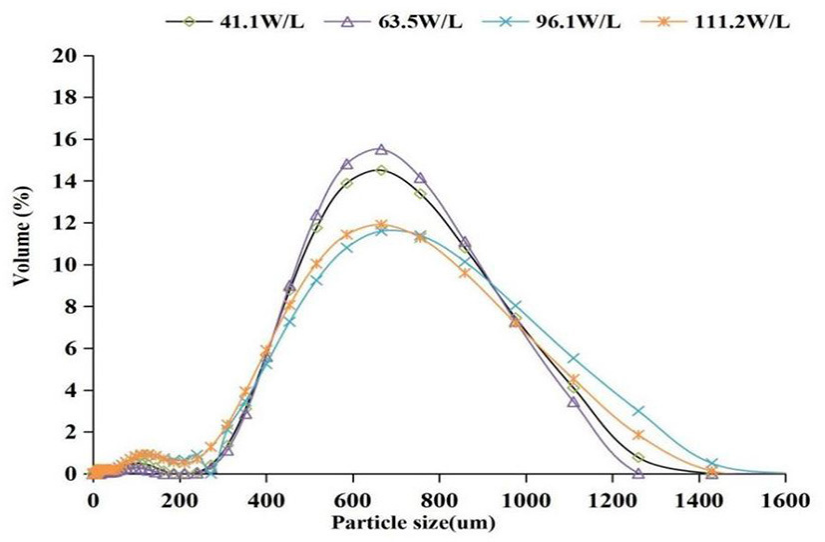
Particle size distribution of grape pomace at different acoustic energy densities.

The extraction process was numerically simulated using the diffusion model to further study the extraction of phenolics in grape pomace by ATPS under ultrasound as well as evaluate the promoting effect of ultrasound. The particle size of grape pomace was determined using a laser particle size analyzer after ultrasonic-assisted extraction with different AED, before simulation. The results are presented in Figure 6. The median particle size (*Dv*50) of grape pomace particles after ultrasound-assisted extraction with different AED at 41.1, 63.5, 96.1, and 111.2 W/L was 671.67, 674.00, 667.00, and 669.00 um, respectively. The findings showed no significant difference in particle size under different ultrasonic conditions (*P* > 0.05). Therefore, particle size change of grape pomace was not considered when constructing the mass transfer diffusion model.

**Figure 7:**
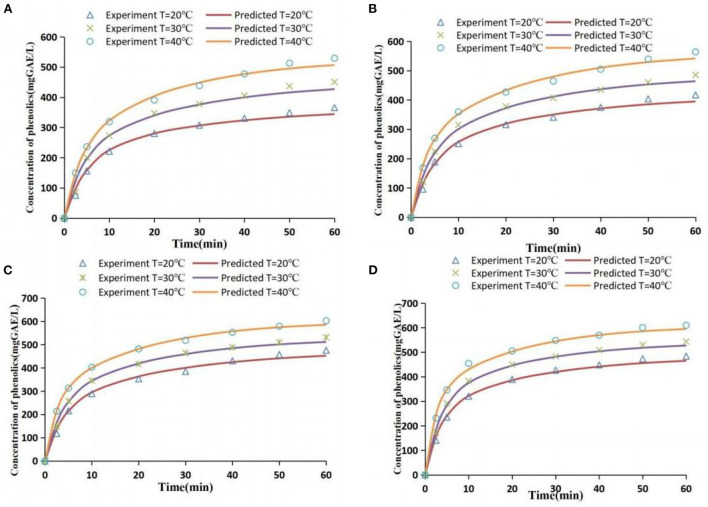
Comparison of experimental values and simulated values [extraction conditions: **(A)** 41.1 W/L; **(B)** 63.5 W/L; **(C)** 96.1 W/L; **(D)** 111.2 W/L].

The values of effective diffusion coefficient *De* under different extraction conditions and results of the evaluation of model fitting degree are presented in Supplementary Table 1. *RMSE* values of all treatment groups were low, *AAD* values were below 10%, and *R*^2^ was above 0.99. This indicates that the diffusion model can effectively fit the mass transfer process of phenolic extraction. A comparison of simulated and experimental values under all extraction conditions is presented in Figure 7. The simulated values were slightly different from the experimental values in some ranges; however, the fit degree was high and the correlation was good.

Effective diffusion coefficient *De* is an important parameter for evaluating mass transfer and diffusion process of phenolics. It indicates the speed of diffusion of the target substance in the medium. The effective diffusion coefficient *De* ranged between 1.67 × 10^−10^ and 5.83 × 10^−10^ m^2^/s under the extraction conditions of this study (Supplementary Table 1). Tao et al. (18) reported that the *De* value ranged from 5.0 × 10^−11^ to 1.58 × 10^−10^ using distilled water as extraction solvent. The *De* value ranged from 1.62 × 10^−10^ to 4.67 × 10^−10^ when the solvent was 50% ethanol-aqueous solution, which is close to the *De* value range obtained in the current study. This indicates that the range of *De* values obtained in the present study is reliable.

*De* value is correlated with material structure, solvent type, and extraction conditions (such as temperature and acoustic energy density). A high temperature is correlated with a high *De* value (*P* < 0.05) under the same AED condition (Supplementary Table 1). This observation may be because the thermal activation energy of atoms is higher and the migration rate increases at a higher temperature, thus increasing the diffusion coefficient (42). This result was consistent with findings from previous research (43). A high AED was correlated with a high *De* value (*P* < 0.05) at the same temperature (Supplementary Table 1). The findings of the present study showed that the particle size of grape pomace did not change significantly under different acoustic energy densities. This indicates that ultrasonic waves to grape pomace mainly damaged the outer tissue of the particles. An increase in AED promotes damage to ultrasonic cavitation effect and mechanical effect on the complete surface of grape pomace particles. In addition, high AED promotes the internal diffusion of polyphenol, thus increasing the *D*e value (18).

The quadratic polynomial function was used in this study to explore the relationship between *De* value and temperature and AED. The results were as shown below:


(14) $$\begin{array}{l} De\times 10^{10}=-1.599+0.02055\cdot AED+0.1213\cdot T\\ \;-6.285\cdot 10^{-5}\cdot AED^{2}-6.25\cdot 10^{-5}\cdot T^{2}\\ \;-7.966\cdot 10^{-5}\cdot AED\cdot T\;\left(R^{2}=0.991\right) \end{array}$$

*R*^2^ of this equation was 0.991, indicating a high fit degree. The function shows that the effect of temperature on the *De* value is higher compared with that of sound energy density, which is consistent with previous results (44).

**Figure 8:**
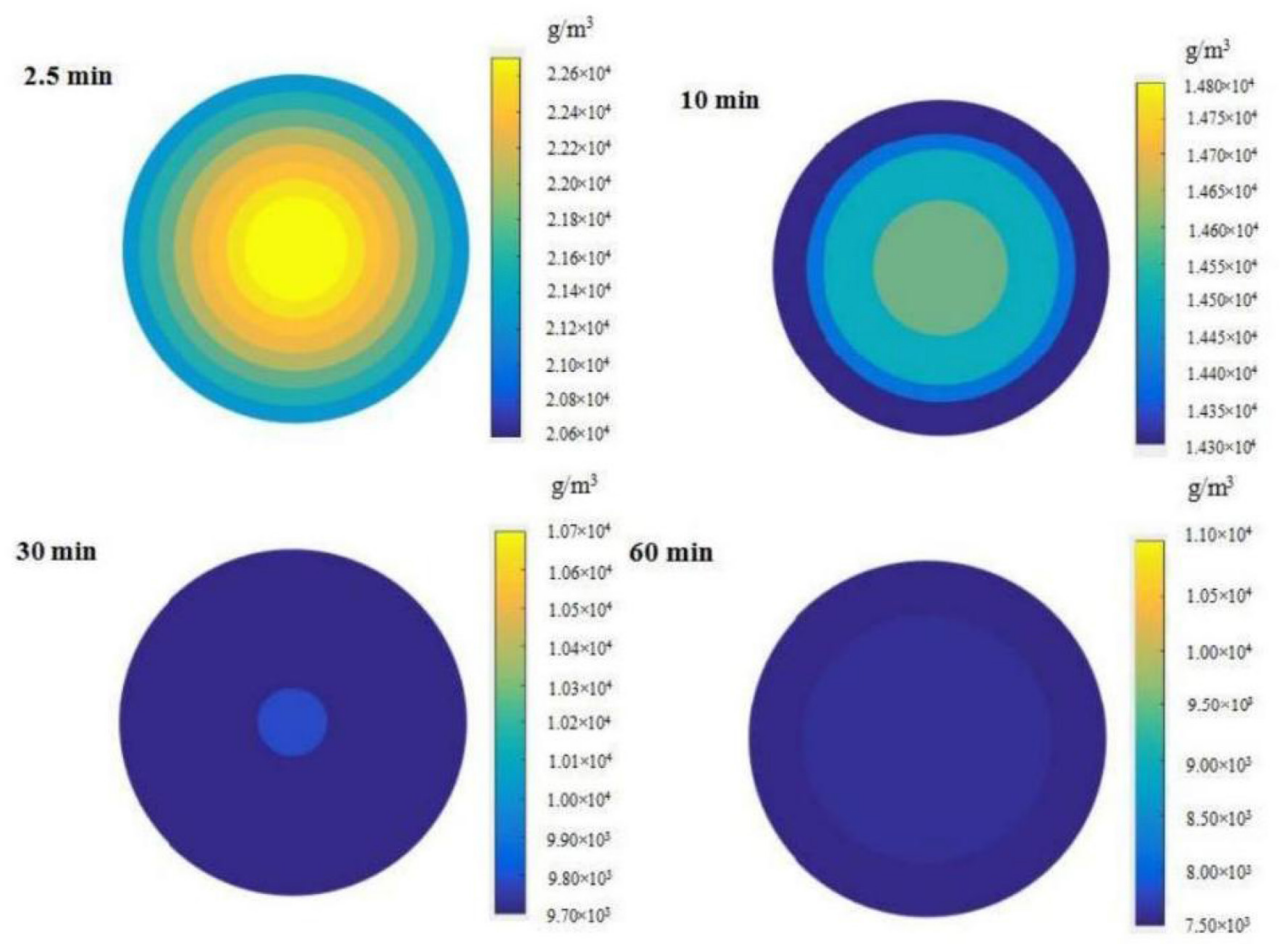
Distribution of phenolics content within grape pomace at different extraction time points (Extraction conditions: 30°C and 96.1 W/L).

The distribution of phenolics in grape pomace was simulated by a programming method based on the diffusion model when the extraction time was 2.5, 10, 30, and 60 min, to explore the change in the distribution of phenolics in grape pomace during extraction. The simulation results at 30°C and 96.1 W/L are presented in Figure 8. The findings showed that between 2.5 and 60 min, the occurrence of particle interior near the surface was associated with lower phenolic content. The phenolic content distribution became more uniform with an increase in extraction time, and the difference between the phenolic content in the core part and the surface became smaller.

### Analysis of individual phenolic content

The yields and the content of individual phenolics in grape pomace (extracted three times) were obtained by ultrasound-assisted ATP extraction (30°C and 96.1 W/L) and reciprocating shaking ATP extraction (30°C and 100 rpm) with 50% ethanol solution crude extraction (30°C and reciprocating shaking) are presented in Table 2. The purpose of analyzing the content of individual phenolics was to analyze the extraction from the perspective of individual phenolics; therefore, only anthocyanins and flavonols were analyzed. The findings showed that the yield of anthocyanins extracted by ultrasound with exception of paeoniflorin pigm-3-glucoside was significantly higher relative to the yield obtained by non-ultrasonic extraction (*P* < 0.05). The findings showed that the ultrasonic method effectively promoted the extraction of anthocyanins. The extraction yield under ultrasonic conditions of the three individual anthocyanins was more than 75% of the total content. The extraction yield of the three individual anthocyanins under non-ultrasonic extraction using 50% ethanol solution was higher compared with that under reciprocating shaking ATP. This is mainly attributed to the different solubilities of anthocyanins due to the different polarities of the two systems and solutions. Yilmaz and Toledo (45) reported that the solvent type significantly affects the extraction rate of phenolics. The extraction rates for rutin using 50% ethanol solution were lower than the rates achieved using ATPS (*P* < 0.05). However, a higher yield of myricetin was obtained using ATPS compared with 50% ethanol solution. This difference in rates can be attributed to different solubilities of rutin and myricetin under different solvent systems.

**Table 2:** Extraction yield of individual anthocyanins and flavonols.

**Species**	**Monomer phenols**	**Ultrasound-assisted ATP extraction** **(μg/g)**	**Reciprocating shaking ATP extraction (μg/g)**	**50% ethanol solution crude extraction** **(μg/g)**	**Raw materials** **(μg/g)**
	Centrinin-3-glucoside	46.47 ± 1.74b	29.76 ± 2.93d	42.12 ± 2.92c	61.28 ± 0.27a
Anthocyanins	Paeoniflorin pigm-3-glucoside	27.01 ± 3.79b	16.35 ± 1.48c	27.02 ± 1.42b	31.86 ± 1.80a
	Mallet pigment−3-glucoside	35.97 ± 2.32b	22.74 ± 2.64d	28.90 ± 2.17c	42.43 ± 2.98a
Flavonols	Rutin	30.80 ± 3.54a	27.78 ± 2.94b	19.40 ± 1.75c	35.04 ± 7.60a
	Myricetin	23.89 ± 1.37c	19.96 ± 1.57c	30.40 ± 0.82b	69.97 ± 3.37a

Values followed by different letters in each line indicate significant differences (*p* < 0.05) among pre-treatments.

### Distribution of sugars at the top and bottom phases during extraction

**Figure 9:**
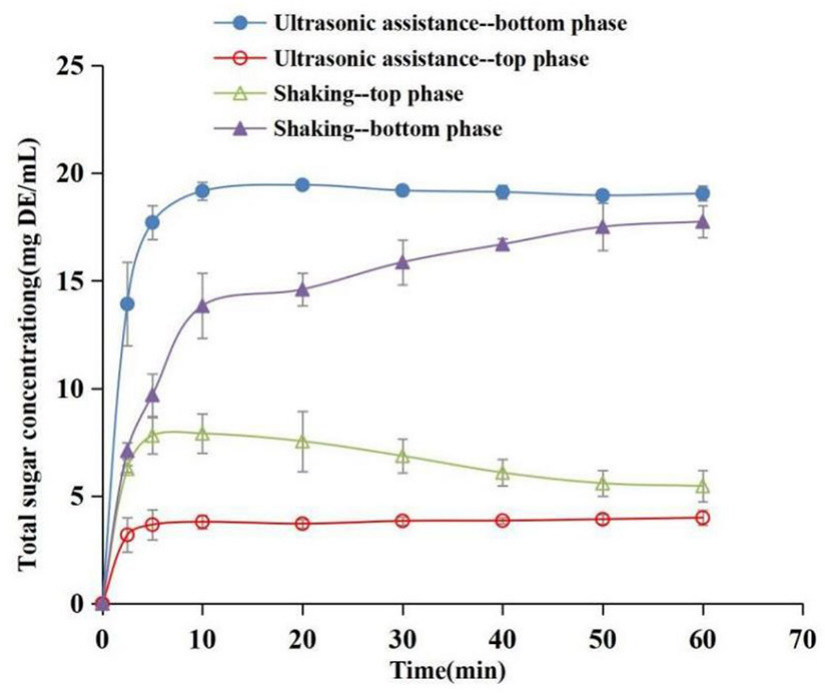
Concentration evolution of total sugars as a function of time in top phase and bottom phase (Extraction conditions: sonication: 30°C and 96.1 W/L; shaking: 30°C and 100 rpm).

The separation effect of ATP on sugar is an important reference index for the purification of phenolics. The concentration evolution of total sugars in the top and bottom phases during the extraction process was determined to evaluate the separation properties of phenolics and sugars (Figure 9). The findings showed that total sugar content increased gradually with an increase in time during the extraction process, and the increasing trend was significant in the bottom phase compared with the top phase. The concentration of sugar in the top and bottom phases under ultrasound exhibited a significant difference at 2.5 min, whereas the difference was not significant under reciprocating shaking. The distribution speed of sugar in ATPS under the ultrasound method was significantly higher than that of the water bath oscillation. In addition, the distribution coefficient under the ultrasound-assisted condition was significantly higher compared with that under the reciprocating shaking condition (*P* < 0.05) at 60 min. The partition coefficients of the top phase sugar contents were 4.80 (ultrasound) and 3.29 (reciprocating shaking), respectively, at 60 min, and the recovery rates were 79.86% (ultrasound) and 72.96% (reciprocating shaking), respectively. This implies that sugars were mainly distributed in the salt-rich phase during ATP extraction of phenolics. Wu et al. (15) separated anthocyanins and sugars using an aqueous two-phase system of ethanol-ammonium sulfate. The results showed that 89.5% of the sugars were distributed in the bottom phase. In the present study, phenolics were mainly distributed in the top alcohol-rich phase; thus the system can effectively separate phenolics and sugars in different phases, aiding the purification of phenolics.

### Comparison of phenolics purity under different extraction conditions

**Figure 10:**
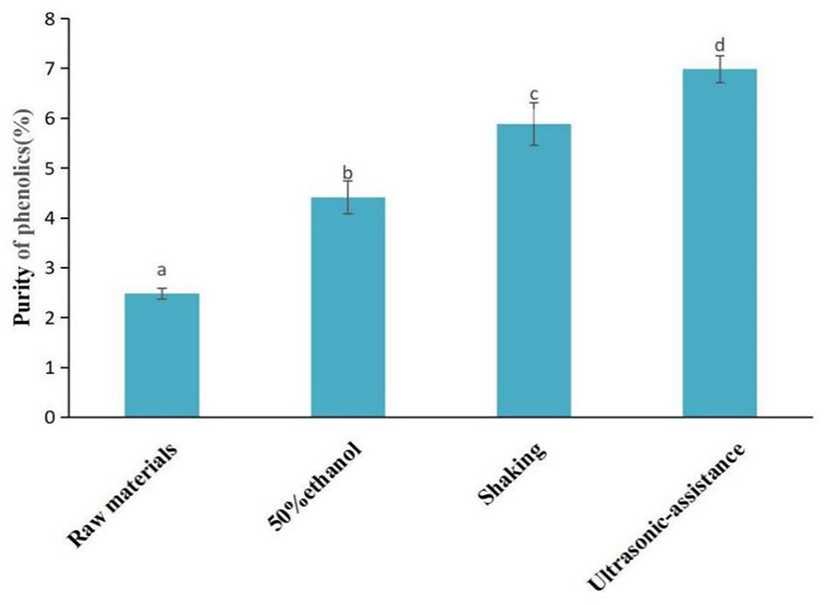
Comparison of purity of phenolics in extracts using different extraction methods.

The purity of phenolics obtained by ultrasonic-assisted ATP extraction (30°C and 96.1 W/L), reciprocating shaking ATP extraction (30°C and 100 rpm), 50% ethanol solution crude extraction (30°C and 100 rpm), and the purity of phenolics in raw materials were compared (Figure 10). The findings showed that the purity of phenolics was 6.98% after ultrasonic-assisted ATP extraction, which was 181.15 and 58.3% higher compared with 2.48% of raw materials and 4.41% of conventional 50% ethanol crude extraction. The purification effect was significantly different among the three methods (*P* < 0.05). In addition to polyphenols, grape pomace contains sugars, organic acids, pectin, oligosaccharides, protein, minerals, vitamins, and other substances (46, 47). The polarity of the top and bottom phases of ATPS is quite different; thus the molecular forces and dissolution properties of these substances in the top and bottom phases are also different. In addition, the compounds are distributed in different phases during the extraction process, which helps to improve the purity of target extraction components.

## Conclusion

The findings of the present study show that ultrasound-assisted ATP is an effective technology for the extraction and purification of phenolics from grape pomace. The appropriate phase composition concentration of ATPS for extraction of phenolics from grape pomace was 30% ethanol (w/w) and 20% ammonium sulfate. The yield of phenolics increased with an increase in AED and temperature. ANN showed high efficacy in correlating extraction parameters with phenolic yield with a correlation coefficient above 0.98. The model showed high efficacy in the visualization and intelligent extraction of phenolics. Moreover, Fick's second law diffusion model revealed the mechanism of ultrasound-assisted ATP in the mass transfer process. Notably, *De* value increased with an increase in AED and temperature. Three anthocyanins and two flavonols were analyzed by HPLC, and the results indicated that the ultrasound method improved the extraction yield of anthocyanins. Higher yields of rutin and myricetin were achieved using ATPS and a 50% ethanol aqueous system, respectively. Sugars were mainly distributed in the bottom phase, whereas phenolics were mainly distributed in the bottom phase. This partition characteristic effectively alleviates the influence of impurities such as sugars on the quality of phenolics. The phenolic purity of the final extract was 6.98%, which was 58.3% higher compared with that of the traditional solid-liquid extraction, indicating effective preliminary purification of phenolics using the method. In summary, enhanced extraction of phenolics and preliminary purification of phenolics was achieved using ultrasound-assisted ATP extraction technology. This strategy has broad application prospects in phenolic extraction from grape pomace and promoting resource utilization of grape pomace.

However, one of the major trends is that there is gaining interest in using new raw materials with benign, eco-friendly, low cost, and recyclable characteristics to form ATPS. Salts such as phosphates and sulfates would pose wastewater treatment problems if the salts are not subjected to recycling. Hence, efforts have been put into the replacement of these salts with biodegradable salt, such as citrate.

## Data availability statement

The original contributions presented in the study are included in the article/Supplementary material, further inquiries can be directed to the corresponding author/s.

## Author contributions

GX: conceptualization, methodology, validation, formal analysis, writing—original draft preparation, and writing—review and editing. JS: software. JL: methodology and writing—review and editing. DL: formal analysis. YT: software, formal analysis, writing—original draft preparation, and supervision. CS: supervision. YH: conceptualization, writing—review and editing, and supervision. All authors have read and agreed to the published version of the manuscript.

## Funding

This work was supported by the Fundamental Research Funds for the Central Universities, China (Grant No. KJQN201824) and the National Natural Science Foundation of China (Grant No. 31701616).

## Conflict of interest

The authors DL, YT, and YH had previously collaborated with the reviewer PS. The remaining authors declare that the research was conducted in the absence of any commercial or financial relationships that could be construed as a potential conflict of interest.

## Correction Note

A correction has been made to this article. Details can be found at: 10.3389/fnut.2025.1697706.

## Publisher's note

All claims expressed in this article are solely those of the authors and do not necessarily represent those of their affiliated organizations, or those of the publisher, the editors and the reviewers. Any product that may be evaluated in this article, or claim that may be made by its manufacturer, is not guaranteed or endorsed by the publisher.
