# Expression of Concern: Simultaneous extraction and preliminary purification of polyphenols from grape pomace using an aqueous two-phase system exposed to ultrasound irradiation: process characterization and simulation

**DOI:** 10.3389/fnut.2024.1450415

**Published:** 2024-07-03

**Authors:** Frontiers Editorial Office

In the published article, the conflict of interest lacked necessary details regarding a previous collaboration between authors Dandan Li, Yang Tao and YongbinHan & reviewer Pau Loke Show. An investigation by the Research Integrity auditing team found that this conflict of interest did not unduly impact the peer review process or scientific validity of the article. As a result, a Correction has been published, and the Expression of Concern is considered resolved. This notice remains published to retain a transparent record.

## Correction note

This article has been corrected with minor changes. These changes do not impact the scientific content of the article.

