# Correction: Simultaneous extraction and preliminary purification of polyphenols from grape pomace using an aqueous two-phase system exposed to ultrasound irradiation: process characterization and simulation

**DOI:** 10.3389/fnut.2025.1697706

**Published:** 2025-09-05

**Authors:** Frontiers Production Office

**Affiliations:** Frontiers Media SA, Lausanne, Switzerland

**Keywords:** ultrasound, aqueous two-phase, grape pomace, phenolic compounds, artificial neural network, diffusion model, purification

In the published article, the conflict of interest lacked necessary details regarding a previous collaboration between authors Dandan Li, Yang Tao and Yongbin Han & reviewer Pau Loke Show. An investigation by the Research Integrity auditing team found that this conflict of interest did not unduly impact the peer review process or scientific validity of the article.

The correct conflict of interest statement appears below

## Conflict of interest

The authors DL, YT, and YH had previously collaborated with the reviewer PS.

The remaining authors declare that the research was conducted in the absence of any commercial or financial relationships that could be construed as a potential conflict of interest.

The original version of this article has been updated.

