# Supplementary material for: Simultaneous extraction and preliminary purification of polyphenols from grape pomace using an aqueous two-phase system exposed to ultrasound irradiation: Process characterization and simulation
Source: Front Nutr. 2022 Nov 14;9:993475. doi: 10.3389/fnut.2022.993475 (PMC9702536; doi:10.3389/fnut.2022.993475)
Supplement: Supplementary file 1 [file Table_1.DOCX]

**Supplementary Table S1.** Effective diffusion coefficients of phenolics from grape pomace under different extraction conditions and the goodness of fit of the diffusion model

| AED (W/L) | Temperature (℃) | *De* (m*^2^*/s) | *R^2^* | *RMSE* | *AAD* (%) |
| --- | --- | --- | --- | --- | --- |
| 41.1 | 20 | 1.67×10^-10^ | 0.994 | 9.810 | 3.089 |
| 41.1 | 30 | 2.83×10^-10^ | 0.992 | 14.204 | 5.327 |
| 41.1 | 40 | 4.00×10^-10^ | 0.996 | 11.733 | 2.839 |
| 63.5 | 20 | 2.33×10^-10^ | 0.994 | 11.138 | 3.038 |
| 63.5 | 30 | 3.33×10^-10^ | 0.995 | 10.907 | 2.829 |
| 63.5 | 40 | 4.50×10^-10^ | 0.997 | 10.569 | 2.031 |
| 96.1 | 20 | 3.17×10^-10^ | 0.994 | 11.874 | 2.952 |
| 96.1 | 30 | 4.33×10^-10^ | 0.998 | 8.480 | 1.702 |
| 96.1 | 40 | 5.42×10^-10^ | 0.999 | 7.508 | 1.382 |
| 111.2 | 20 | 3.67×10^-10^ | 0.996 | 9.775 | 1.655 |
| 111.2 | 30 | 4.83×10^-10^ | 0.997 | 9.140 | 1.72 |
| 111.2 | 40 | 5.83×10^-10^ | 0.997 | 10.776 | 1.554 |


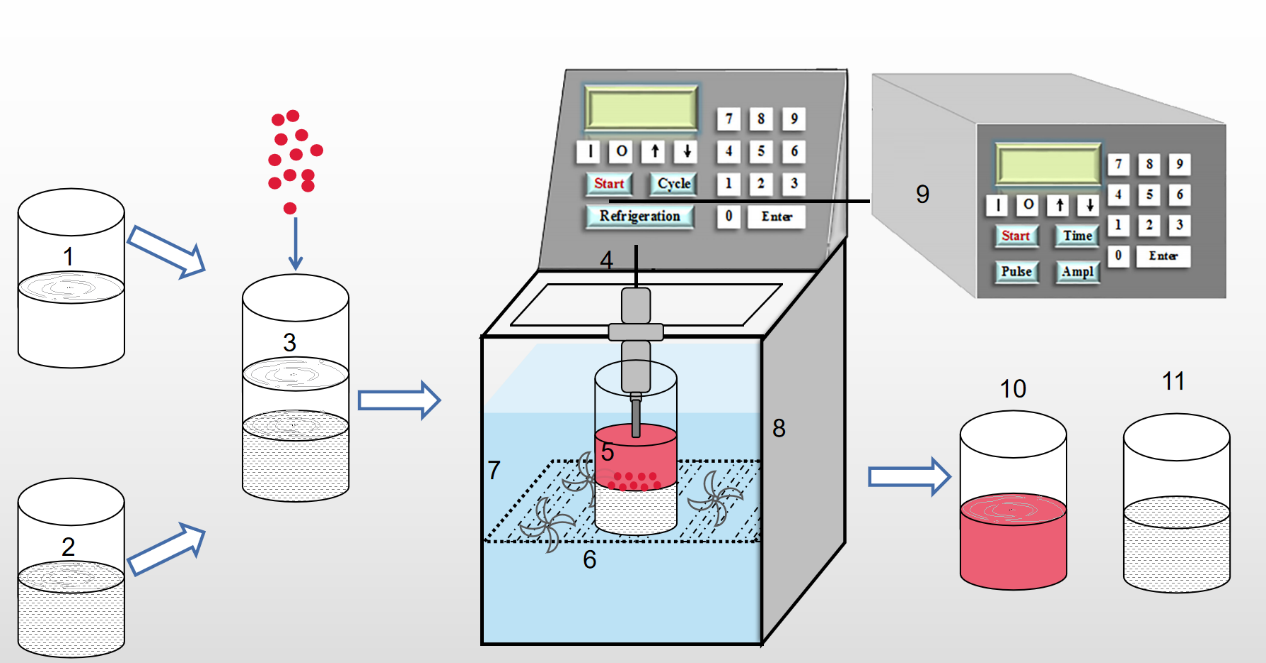


**Supplementary Fig S1.** Schematic diagram of ATP assisted simultaneous extraction and purification.1:Anhydrous; 2:Ammonium sulfate solution with a certain concenteration; 3:Grape pomace; 4: Ultrasonic probe; 5:Water; 6:Propeller; 7:Electro-thermostatic Water Cabinet; 8:Generator; 9:Top phase; 10:bottom phase.


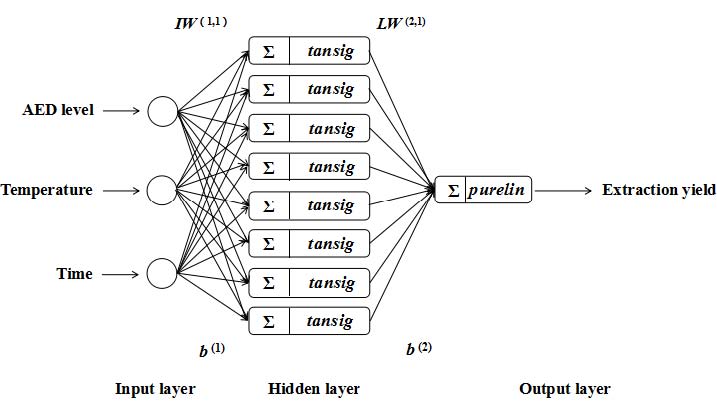


**Supplementary Fig S2.** ANN model for evaluating the relationship between extraction yield of phenolics and AED level, temperature and time.


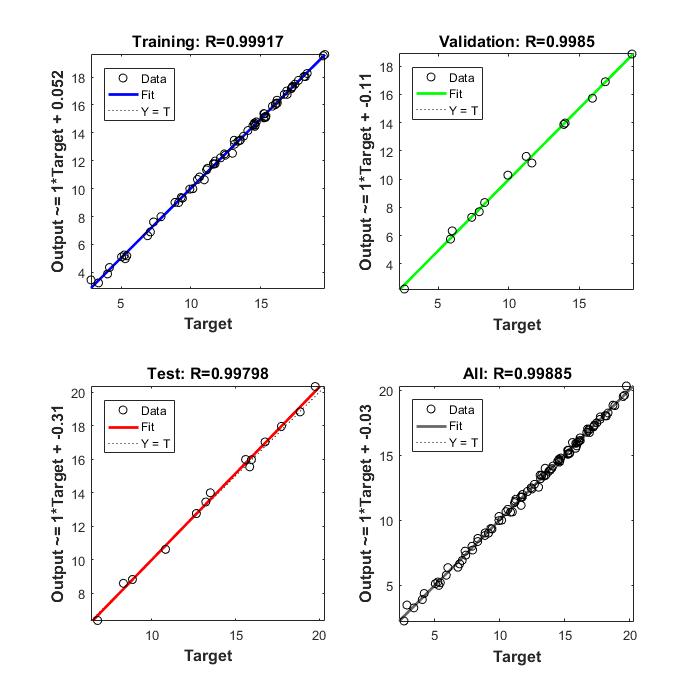


**Supplementary Fig S3.** Regression of prediction values of the artificial neural network model.
